# Fixed Dose versus Height-Adjusted Conventional Dose of Intrathecal Hyperbaric Bupivacaine for Caesarean Delivery: A Prospective, Double-Blinded Randomised Trial

**DOI:** 10.3390/jcm9113600

**Published:** 2020-11-08

**Authors:** Katarzyna Białowolska, Bartosz Horosz, Agnieszka Sękowska, Małgorzata Malec-Milewska

**Affiliations:** 1 Department of Anaesthesiology and Intensive Care, Medical Centre of Postgraduate Education, Orlowski Hospital, Ul. Czerniakowska 231, 00-416 Warsaw, Poland; kbialowolska@cmkp.edu.pl (K.B.); mmalec@cmkp.edu.pl (M.M.-M.); 2 2nd Department of Obstetrics and Gynaecology, Medical Centre of Postgraduate Education, Bielanski Hospital, Ul. Cegłowska 80, 01-809 Warsaw, Poland; asekosia@poczta.onet.pl

**Keywords:** elective caesarean, spinal anaesthesia, hyperbaric bupivacaine, hypotension

## Abstract

The optimal intrathecal dose of local anaesthetic for caesarean section (CS) anaesthesia is still being debated. We performed a study to compare the effectiveness and safety of spinal anaesthesia with 12.5 mg of hyperbaric bupivacaine and a dosing regimen of conventional doses adjusted to parturient height. One hundred and forty parturients scheduled for elective CS were enrolled. The fixed-dose group (FD) received a spinal block with 12.5 mg of hyperbaric bupivacaine with fentanyl, whereas the adjusted-dose group (AD) received a height-adjusted dose of bupivacaine (9–13 mg) with fentanyl. Sensory block ≥ T5 dermatome within 10 min and no need for supplementary analgesia were set as the composite primary outcome (success). Rates of successful blocks and complications were compared. Complete data were available for 134 cases. Spinal anaesthesia was successful in 58 out of 67 patients in the FD group and 57 out of 67 in the AD group (*p* > 0.05). Eight spinals in each group failed to produce a block ≥ T5 in 10 min, and one patient in the FD group and two in the AD group required i.v. analgesics despite sensory block ≥ T5. No differences were noted in terms of hypotension, bradycardia and nausea between the FD and AD groups. Compared to the height-adjusted dose regimen based on conventional doses of hyperbaric bupivacaine, the fixed dose regimen of 12.5 mg was equally effective and did not increase the risk of spinal block-related complications.

## 1. Introduction

Spinal block is the most common type of anaesthesia used for caesarean section (CS), with nearly 95% of elective and 45% of emergency procedures performed under spinal or spinal-epidural anaesthesia [1,2]. Due to pregnancy-related cardiovascular changes and required high block levels, the risk of spinal block-related complications in full-term parturients is substantial, of which hypotension, bradycardia and nausea are of major concern [3].

The intrathecal dose of hyperbaric bupivacaine effective in 95% (ED95) of spinals for caesarean section has been established to be 11.2 mg [4], 0.06 mg/cm of height [5], or even more recently 12.6 mg of hyperbaric bupivacaine [6].

Limiting the intrathecal dose of local anaesthetic (LA) to doses as low as 6–7 mg of hyperbaric bupivacaine has been proposed in order to minimize the complication rate, but this comes at an increased risk of inadequate anaesthesia and thus may require the use of combined spinal-epidural technique to assure the desired level of effectiveness [7,8,9].

Dose-adjustment protocols were proposed for conventional doses, with the main interest in limiting the rate of spinal block-related maternal hypotension. Despite convincing data on no relation between intrathecal spread of local anaesthetic and maternal height and weight, adjusting the dose of LA to patient size is still being debated [10,11,12,13,14,15].

Whether it is clinically indicated to adjust the doses nearing ED95 of heavy bupivacaine to maternal size remains unclear.

The standard protocol in our centre is based on relatively high doses of hyperbaric bupivacaine (close to ED95), which are adjusted to parturient height.

This study was designed to compare the outcomes of spinal anaesthesia performed with 12.5 mg of hyperbaric bupivacaine with 25 μg of fentanyl to a dosing regimen incorporating conventional doses of bupivacaine (9–13 mg) adjusted to parturient height with 25 μg of fentanyl. We hypothesized that using a fixed dose of 12.5 mg hyperbaric bupivacaine would result in better effectiveness than a height-adjusted dose of hyperbaric bupivacaine, without increasing the rate of hypotension.

## 2. Materials and Methods

### 2.1. Study Design and Participants

This parallel, randomised, double-blinded study with a planned allocation ratio of 1:1 was performed between July 2017 and July 2019 in the 1st and 2nd Department of Obstetrics and Gynaecology and the Department of Anaesthesiology and Intensive Care, Centre of Postgraduate Education, Bielanski Hospital and Orlowski Hospital, Warsaw, Poland. This report remains in accordance with the Consolidated Standards of Reporting Trials (CONSORT) guidelines on reporting parallel group randomised trials and was registered in ClinicalTrials.com: NCT 03231436. The Study protocol was approved by the institutional Ethics Committee on 12th July 2017 (65/PB/2017). All enrolled subjects provided written informed consent to participate in the study.

Healthy, full-term (gestation > 37 weeks) parturients over 18 years of age with a singleton pregnancy scheduled for elective CS were enrolled in the study. In order to minimize potential bias related to coexisting comorbidities, strict exclusion criteria were applied as follows: absolute contraindications to neuraxial anaesthesia, history of three or more CS’s, American Society of Anesthesiologists (ASA) physical status > 2, body mass index (BMI) > 35 kg/m^2^. Women with any known cardiovascular comorbidity were also excluded (e.g., primary hypertension, pregnancy-induced hypertension, any form of arrhythmia), as well as women in active labour, with intrauterine growth retardation (IUGR), known foetal abnormalities and increased risk of postpartum haemorrhage (e.g., placenta accreta/percreta).

### 2.2. Blinding

Sequentially numbered sealed envelopes containing hyperbaric bupivacaine dose descriptions according to allocated group were handed to the anaesthetist performing the spinal block prior to the beginning of the procedure. Blinding was ensured by the presence of two anaesthetists: one not blinded to the group allocation, who prepared the dose for spinal injection and performed the spinal block and the second blinded to the group allocation, who was in charge of patient preparation for anaesthesia in the operating room (OR) and for the procedure after the block was placed, but who was not present during dose preparation and intrathecal injection.

### 2.3. Perioperative Care, Study Intervention and Data Acquisition

All patients fasted for 6 h and received an infusion of 1000 mL crystalloids within an hour prior to the procedure, 50 mg of ranitidine, 10 mg of metoclopramide and 2 g of cefazoline. After admission to the operating room (OR), routine monitoring was applied (blood pressure, pulse oximetry, continuous electrocardiogram) along with an intravenous infusion of 500 mL of balanced crystalloid (co-loading). At this point every parturient was informed on possible symptoms which could be experienced after the block placement and during the procedure, with an emphasis on pain, nausea and vomiting. Throughout the surgery patients were frequently asked about these symptoms and advised to report even mild nausea and pain, even if considered insignificant.

Spinal anaesthesia was performed in the seated position, in the L3/4 or L4/5 interspace, judged by Tuffier’s line, using a 27 gauge pencil point spinal needle with an introducer, with the needle orifice directed laterally during intrathecal injection. After the injection, the patient remained seated for 10–15 s and was then placed supine with 15 degrees of left lateral tilt. The fixed dose group received spinal anaesthesia with 12.5 mg (2.5 mL) of 0.5% hyperbaric bupivacaine and 25 μg of fentanyl (0.5 mL), whereas the adjusted dose group received a height-adjusted dose (Table 1) and 25 μg of fentanyl (0.5 mL).

Sensory loss to cold (ethyl chloride spray) was determined bilaterally every 2 min for the first 10 min, then after 20, 30 and 40 min. For assessment of motor block a modified Bromage scale was used (0: no motor block, 1: inability to raise extended legs, 2: inability to flex knees, able to move feet, 3: inability to do dorsiflexion of foot, 4: inability to move at all). Surgery commenced once sensory block ≥T5 was achieved and complete motor block was established (Bromage score 4). Both time to reach T5 level of block and time to surgical incision were recorded. Following delivery, 5 units of oxytocin or 100 μg of carbetocin were administered i.v. and the operating table was returned to neutral position. If the patient reported significant discomfort or pain after the incision and prior to delivery, single dose of ketamine and propofol was administered as rescue medications. If not sufficient, conversion to general anaesthesia was planned. After delivery 50 μg of fentanyl was given i.v. as first choice, followed by ketamine and propofol if necessary. Intravenous anaesthetics or analgesics administered in the OR both intraoperatively and postoperatively were recorded and reported as the need for supplemental analgesia.

Demographic variables recorded prior to procedure were age, weight, height, weight gain during pregnancy, parity and gestational age. Neonatal weight and Apgar scores were acquired and recorded after delivery.

Automated, noninvasive blood pressure measurements were taken twice before the spinal block, immediately after block placement and afterwards every three minutes. The lower of the two pre-spinal mean arterial pressures (MAP) and systolic blood pressures (SBP) were considered baseline. Hypotension was defined as a > 30% drop in MAP from baseline or SBP < 85 mmHg at any point during the procedure and was treated with ephedrine 5 mg, repeatedly if necessary. Heart rate < 60/min was considered bradycardia, which was recorded and treated with i.v. 0.6 mg of atropine. Intraoperative fluid management consisted of balanced crystalloids at the discretion of the responsible anaesthetist. Incidence of hypotension, bradycardia and a total dose of ephedrine administered were recorded. All episodes of nausea and vomiting were recorded until the patient was transferred to the recovery room. Intensity of nausea was not graded, while both retching and vomiting were recorded and analysed as vomiting. Postoperative pain management consisted of 1g of paracetamol every 6 h and 10 mg subcutaneous morphine every 3 h when pain on the numeric rating scale (NRS) > 3, and an i.v. morphine top-up if required, at the discretion of anaesthetist in charge. Data on the time from spinal block to first postoperative opioid requirement and the total amount used were retrospectively obtained from recovery room charts.

### 2.4. Primary and Secondary Outcomes

Our primary outcome was successful spinal block, defined as sensory loss to cold in the ≥ T5 dermatome within 10 min of intrathecal injection and with no need for supplemental analgesia throughout the procedure.

Secondary endpoints were the occurrence of complications (hypotension, bradycardia, nausea, vomiting), time from spinal injection to first dose of opioid and postoperative opioid consumption (total dose of morphine in 24 h after surgery).

### 2.5. Sample Size and Statistical Analysis

Sample size calculation revealed that 63 subjects were required in each group for the study to detect a 15% difference in success ratio with 90% power and a significance level of α = 0.05. Considering the reported differences in effectiveness between low and standard doses of bupivacaine, we assumed a 15% difference to be of clinical significance [9]. On-line randomisation using block randomisation method was performed with a 1:1 allocation ratio for 140 cases, allowing for a number of drop-outs.

Nominal variables are presented as *n* (%), and continuous variables as a mean (±SD) or median (IQR), depending on the distribution of data. Continuous variables were compared with the use of independent samples *t-*test or Mann-Whitney U test. Dichotomous variables were analysed with the χ^2^ test or Fisher exact test, as appropriate. Relative risk (RR) was calculated for nominal and differences in means or differences in medians (MD) for continuous variables, with 95% confidence interval (CI). To allow for RR calculations in cases of zero incidence, 0.5 was added to all ingredients of equation. All tests were two-tailed, and differences were considered significant at the level of *p* < 0.05. Analyses were performed with statistical software R version 3.5.1 (www.r-project.org), R Foundation for Statistical Computing, Vienna, Austria.

## 3. Results

**Figure 1:**
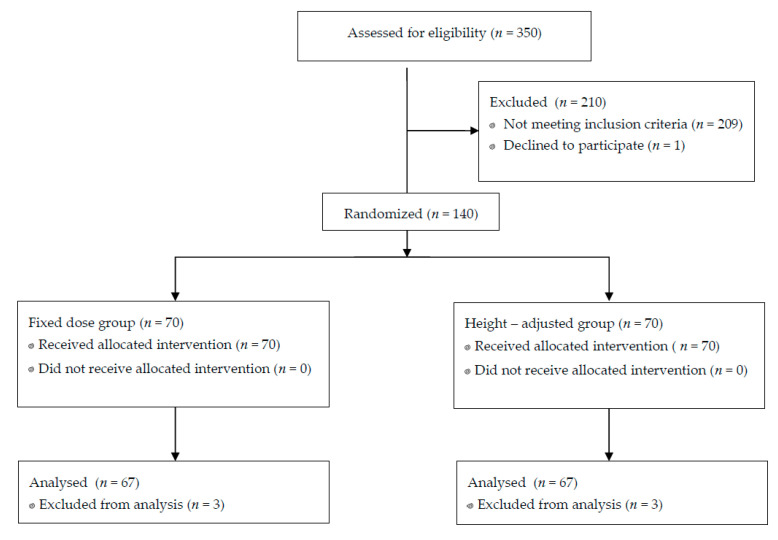
Consolidated Standards of Reporting Trials (CONSORT) flow diagram.

Between July 2017 and July 2019, 350 women were assessed for eligibility of whom 141 met the inclusion criteria. A total of 140 patients was enrolled, 70 in each group. One parturient declined to participate in the study. The CONSORT flow diagram is presented in Figure 1. Three patients in each group were excluded from analysis: in four cases (two in each group), there was no clinical sign of spinal blockade after intrathecal injection and the procedure had to be repeated. One case in each group was excluded due to protocol violation (sedation administered for the reason other than block failure—adjusted dose (AD) group; inability to assume fully supine position following intrathecal injection—fixed dose (FD) group).

A total of 134 cases were finally analysed. Demographic data, baseline obstetric characteristics and neonatal outcomes were no different between the groups, except for bupivacaine doses, which were significantly lower in the AD group (Table 2). The study population consisted of relatively tall women: there were only 3 patients ≤ 155 cm of height in each group. All parturients were Caucasian except for one, who was Asian.

### 3.1. Primary Outcome

There was no difference in the number of successful spinal blocks between the groups as defined by study protocol, with a success rate of 86.6% in the FD group and 85.1% in the AD group (Table 3). Due to a pre-defined primary endpoint, all spinals that failed to produce a block level of at least T5 in 10 min following intrathecal injection were considered failures, regardless of the final block level or further requirement of additional analgesia. Eight patients in each group fulfilled this criterion. Only one patient in the FD group and two in the AD group required supplemental analgesia despite sensory block of ≥ T5 at 10 min. Of patients with spinal block below T5 at 10 min two patients in FD group and none in AD group required systemic analgesia during the procedure.

The distribution of the final level of sensory blockade was also similar in both groups, and the vast majority of blocks reached the level of T4 or higher (Table 3).

The highest observed level of block was T1, which was seen in four patients in the AD group and two patients in the FD group. Ketamine, propofol and fentanyl (added after delivery) were required in three cases (inadequate block before delivery: two in the FD group and one in the AD group), whereas i.v. fentanyl was sufficient in two cases (one in each group); no conversions to general anaesthesia were necessary.

### 3.2. Secondary Outcomes

No significant differences were noted in terms of secondary endpoints. Spinal block-related complications and postoperative opioid consumption are summarised in Table 4.

The rate of complications was similar in both groups, of which bradycardia and vomiting occurred in the FD group only. Bradycardia was effectively treated with a single dose of atropine 0.6 mg, while nausea and vomiting episodes were one-off events and no specific treatment was necessary.

There was no difference in hypotension rate between the groups. Of interest, none of the three women less than 155 cm in height in the FD group and two in the AD group required vasopressor for hypotension.

Five patients in the FD group and one in the AD group did not require postoperative opioids, while neither time to first dose nor total amount of morphine given during 24 h after surgery differed between the groups.

## 4. Discussion

This study showed that using a fixed dose of 12.5 mg of hyperbaric bupivacaine, regardless of parturient height, resulted in a similar success and complication rate when compared to a height-adjusted regimen (9–13 mg) in a population of fairly tall patients.

The quest for the ideal intrathecal dose of local anaesthetic has been ongoing for decades, since atraumatic spinal needles were introduced and neuraxial blocks became the most common mode of anaesthesia for caesarean section [16].

The primary end-point of this study was established to assess clinical effectiveness of the compared regimens. For this reason, it was defined as both successful anaesthesia at 10 min following intrathecal injection and no need for supplemental analgesics during the procedure. No differences were found between the groups in this regard. As spinal blockade may need different lengths of time to reach its maximal level, the time used to define success in clinical trials varies between 10 min [4,12,17,18], 15 min [19], or even 20 min [5,20].

Although longer spinal-to-incision times are not regarded as harmful to the parturient, some data indicate that it may pose a threat to neonatal wellbeing [21]. In urgent cases the time constraint may become one of the most important factors.

Using conventional doses of LA and assuming a high success rate, we did not use epidural catheters as part of the anaesthetic plan, which is a common practice in studies on the effectiveness of low dose spinal anaesthesia, and may prolong the time needed to perform the block [16,22].

To the best of our knowledge, this is the first trial where the effectiveness of anaesthesia was set as a primary end point and doses near the ED95 of hyperbaric bupivacaine were used in both groups. Despite high doses of bupivacaine, the number of high blocks (maximum level T2, T1) was similar in both groups and none reached cervical levels. Higher block levels were reported with lower and even much lower doses [13,23].

Many factors could contribute to this outcome: body positioning during the procedure (sitting), the level of injection (L3/4 or L4/5), body composition (mean height > 165 cm in both groups) and, less likely, the fact that patients remained in a seated position for 10–15 s following intrathecal injection [24].

The success rate was high, and the time needed to achieve the required level of block was short in both groups, with no statistical differences between them in this regard. This implies that adjusting the dose in range used in this study (9–13 mg) results in a similar success ratio to using a fixed dose of 12.5 mg of hyperbaric bupivacaine.

Despite obvious advantages over other types of anaesthesia, it is impossible to ignore complications caused by intrathecal administration of local anaesthetics in full-term parturients, of which hypotension is the most frequent and may affect maternal and neonatal outcomes. No statistical differences were found between the groups in the frequency of hypotension, bradycardia, nausea and vomiting.

The rate of hypotension was high and use of vasopressor was necessary in more than half of the patients in both groups, which is not uncommon when compared to results of other trials. Minimizing the risk of hypotension was the main purpose of previously proposed dose-adjusting strategies [13,14,25].

However, as the definition of hypotension varies greatly between trials, it is difficult to compare their results [26]. The criteria for hypotension used in our study assumed a quite low absolute SBP (<85 mmHg) and a significant drop in MBP from baseline (>30%). It may be noted that the baseline SBP of patients was rather high (>130 mmHg). No differences were found in the rate of hypotension between groups, which is in contrast to the results of a benchmark study by Harten et al., where dose adjusting (6.5–12 mg) was according to a two-factor regimen (height and weight), reducing the incidence of hypotension when compared to a fixed dose of 12 mg bupivacaine [13]. In a study by Alam et al., where the same two-factor adjustment strategy was compared with a fixed dose regimen of 10 mg, no significant differences were found regarding effectiveness and hypotension ratio between groups [14].

Weight was not included as a factor determining dosage in our dose-adjusted group. The influence of body weight on the subarachnoid spread of local anaesthetic has been debated but is still not clear which factor most contributes to this [12,19]. Although obesity does not appear to influence the ED95 dose, it is suggested that higher BMI may increase the risk of spinal-induced hypotension [27].

Incidence of nausea and vomiting was similar, while vomiting was noted in the FD group only. It is worthy of note that the frequency of maternal nausea and vomiting was low. Similar rates were reported in studies where low doses of LA were used [14,28]. Therefore, the results of our study do not support the notion that adjusting conventional doses of bupivacaine to patient’s height will prevent nausea and vomiting. Similarly, there were no statistical differences in neonatal outcomes, time to first opioid request nor the quantity of morphine used postoperatively between groups.

There are some limitations to this study. Firstly, this study was designed to detect a difference in effectiveness of 15%. It may be argued that even a lower difference might be of significance, but in order to detect this larger trials would be required. Secondly, our study population consisted of healthy, rather tall, and lean women. Since a vital part of the study protocol relied on dose adjustment according to height, patients with extremes of height were not excluded. BMI of the study population was limited to < 35 in order to avoid the potential confounding variable of obesity and achieve a reasonably homogenous group in terms of body composition. Therefore, our results are not representative for all full-term pregnant women.

Thirdly, the difference in complication rate this study was able to detect was not pre-defined and results regarding maternal hypotension naturally depend on the criteria used for its assessment. Our criteria assumed a quite significant decrease in blood pressure. It is not possible to rule out the possibility of different results if other, more strict cut-off values would have been used for defining spinal-induced hypotension, both in terms of incidence and differences between the groups. Further complication-targeted studies may be required to focus on the safety of the compared strategies.

## 5. Conclusions

To conclude, our study showed that adjusting conventional doses of hyperbaric bupivacaine (in the range of 9–13 mg) in combination with fentanyl to the patient’s height results in a similar success ratio and risk of spinal block-related complications when compared to a fixed dose of 12.5 mg bupivacaine with fentanyl in non-obese, fairly tall women. Further studies may be needed to explore safety and clinical application of the compared strategies in parturients of different body habitus.

## Figures and Tables

**Table 1:** Height-adjusted bupivacaine doses used in adjusted-dose (AD) group.

Height	Dose of Hyperbaric Bupivacaine in AD Group	
cm	mL	mg
150–155	1.8	9
156–160	2.0	10
161–165	2.2	11
166–170	2.4	12
>170	2.6	13

**Table 2:** Demographic, obstetric and procedure-related data.

	FD Group (*n* = 67)	AD Group (*n* = 67)	*p*
Age, years	34.0 (32.0–36.0)	33.0 (30.0–36.0)	0.514
Weight, kg	75.73 ± 9.30	78.80 ± 12.17	0.104
Height, cm	165.97 ± 5.83	166.24 ± 6.03	0.794
Body mass index, kg/m^2^	27.49 ± 3.08	28.44 ± 3.60	0.103
Pregnancy weight gain, kg	14.0 (10.0–17.0)	13.0 (10.0–15.5)	0.675
Parity	2.0 (1.0–2.0)	2.0 (1.0–2.0)	0.667
Gestational age, weeks	39.0 (38.0–39.0)	39.0 (38.0–39.0)	0.198
Baseline systolic blood pressure (SBP), mmHg	134.0 (127.0–143.0)	135.0 (124.5–142.0)	0.824
Baseline mean arterial pressure (MAP), mmHg	103.53 ± 10.91	104.17 ± 12,33	0.751
Baseline heart rate (HR), beats/min	94.03 ± 14.96	96.21 ± 14.46	0.393
Time to reach T5 dermatome, min	5.0 (5.0–10.0)	5.0 (5.0–10.0)	0.567
Time to incision, min	8.39 (±2.28)	8.58 (±2.24)	0.621
Neonatal weight, g	3444.7 ± 500.6	3461.2 ± 426.2	0.842
Apgar score	10.0 (10.0–10.0)	10.0 (10.0–10.0)	0.136
Bupivacaine dose, mg	12.5 (12.5–12.5)	11.0 (11.0–13.0)	<0.001

Data are presented as mean (± standard deviation) or median (interquartile range). FD-fixed dose; AD -adjusted dose.

**Table 3:** Anaesthetic outcomes in study groups and height distribution.

	FD Group (*n* = 67)	AD Group (*n* = 67)	RR (95% CI)	*p*
Primary outcome measures				
Number of successful blocks	58 (86.6)	57 (85.1)	1.02 (0.89; 1.17)	>0.999
Block level <T5 at 10 min	8 (11.9)	8 (11.9)	1.00 (0.39; 2.50)	>0.999
Supplemental analgesia required	1 (1.5)	2 (3.0)	0.5 (0.05; 5.38)	>0.999
Highest block levels in groups assessed				
T1	2 (3.0)	4 (6.0)	0.50 (0.09; 2.63)	0.680
T2	17 (25.4)	9 (13.4)	1.89 (0.91; 3.93)	0.126
T3	19 (28.4)	16 (23.9)	1.19 (0.67; 2.10)	0.694
T4	25 (37.3)	34 (50.7)	0.73 (0.50; 1.09)	0.164
T5	3 (4.5)	2 (3.0)	1.50 (0.26; 8.69)	>0.999
T6	1 (1.5)	2 (3.0)	0.50 (0.05; 5.38)	>0.999
Height distribution in study groups				
150–155 cm	3 (4.4)	3 (4.4)	*n*/a	>0.999
156–160 cm	8 (11.9)	10 (14.9)	*n*/a	0.801
161–165 cm	24 (35.8)	22 (32.8)	*n*/a	0.857
166–170 cm	22 (32.8)	15 (22.4)	*n*/a	0.249
>170 cm	10 (14.9)	17 (25.4)	*n*/a	0.195

Data presented as *n* (%) and RR (95% CI). RR-relative risk; CI-confidence interval; FD-fixed dose; AD-adjusted dose. *n*/a: not applicable.

**Table 4:** Secondary outcomes. Data presented as *n* (%) or median (interquartile range).

	FD Group (*n* = 67)	AD Group (*n* = 67)	RR/MD (95% CI)	*p*
Hypotension	40 (59.7)	36 (53.7)	1.11 (0.83; 1.50)	0.655
Bradycardia	2 (3.0)	0 (0.0)	5.00 (0.24; 102.23)	0.496
Nausea	19 (28.4)	14 (20.9)	1.36 (0.74; 2.48)	0.423
Vomiting	3 (4.5)	0 (0.0)	7.0 (0.36; 132.96)	0.244
Ephedrine, mg	0.0 (0.0–10.0)	0.0 (0.0–5.0)	0.00 (-0.00002; 0.00001)	0.601
Time to first opioid requirement, min	210.0 (166.3–290.8)	220.5 (166.3–270.0)	−10.50 (−27.00; 35.00)	0.813
Total morphine, mg	20.0 (10.0–20.0)	20.0 (10.0–20.0)	0.00 (−0.00007; 0.000007)	0.109

RR-relative risk; MD-median difference; CI-confidence interval. FD-fixed dose; AD-adjusted dose.
